# Darunavir/cobicistat showing similar effectiveness as darunavir/ritonavir monotherapy despite lower trough concentrations

**DOI:** 10.1002/jia2.25072

**Published:** 2018-02-12

**Authors:** Alicia Gutierrez‐Valencia, Maria Trujillo‐Rodriguez, Tamara Fernandez‐Magdaleno, Nuria Espinosa, Pompeyo Viciana, Luis F López‐Cortés

**Affiliations:** ^1^ Enfermedades Infecciosas Microbiología y Medicina Preventiva Instituto de Biomedicina de Sevilla/Hospital Universitario Virgen del Rocío/CSIC/Universidad de Sevilla Sevilla Spain

**Keywords:** Darunavir, cobicistat, ritonavir, monotherapy, pharmacokinetic, C_trough_

## Abstract

**Introduction:**

When darunavir (DRV) 800 mg is boosted with 150 mg cobicistat (DRV_cobi_), DRV trough concentration (C_trough_) is about 30% lower as compared to 100 mg ritonavir (DRV_rtv_). DRV_cobi_ shows similar virological efficacy as DRV_rtv_ when combined with two nucleos(t)ide analogue reverse‐transcriptase inhibitors, but it is unknown whether a lower DRV C_trough_ would undermine the effectiveness of DRV_cobi_ when given as monotherapy (mtDRV_cobi_).

**Methods:**

Prospective observational study on virologically suppressed HIV‐infected subjects who switched to mtDRV_cobi_. Virological failure was defined as two consecutive HIV‐RNA >200 copies/mL. Efficacy was evaluated by intention‐to‐treat (ITT) and on‐treatment (OT) analyses, and compared with data from a previous cohort of subjects on mtDRV_rtv_ conducted at our centre. Plasma DRV C_trough_ was measured using LC–MS/MS.

**Results:**

A total of 234 subjects were enrolled. At week 96, the efficacy rates were 67.8% (CI_95_, 61.8 to 73.7) by ITT and 86.9% (CI_95_, 78.0 to 87.7) by OT analyses. The corresponding rates in our historical DRV_rtv_ controls were 67.6% (CI_95_, 60.0 to 75.2) and 83.6% (CI_95_: 77.2 to 90.0). A total of 135 DRV determinations were performed in 83 subjects throughout the follow‐up period, with a median plasma DRV C_trough_ of 1305 ng/mL (range, 150 to 5895) compared with 1710 ng/mL (range, 200 to 3838) in subjects on monotherapy with DRV_rtv_ (*p *=* *0.05).

**Conclusions:**

DRV C_trough_ was lower in HIV‐infected subjects receiving DRV_cobi_ than with DRV_rtv_. However, this did not appear to influence the efficacy of DRV_cobi_, when administered as monotherapy.

## 1 Introduction

Cobicistat is a potent and selective human CYP3A inhibitor without anti‐HIV activity showing a lower potential for undesirable drug‐drug interactions when compared to ritonavir (rtv) 1. When darunavir (DRV) 800 mg was boosted with cobicistat 150 mg (DRV_cobi_) once daily in healthy volunteers, DRV exposure was within the limits of bioequivalence for *C*_max_ and AUC_24h_ compared to DRV_rtv_, but DRV C_trough_ were about 30% lower 2. This difference may not be clinically relevant for combined antiretroviral therapy (cART) since DRV_cobi_ has shown similar virological efficacy as DRV_rtv_ when administered in combination with two nucleos(t)ide analogue reverse‐transcriptase inhibitors (NRTIs) 3, 4. However, it remains unclear whether this is also true for DRV_cobi_ when given as monotherapy (mtDRV_cobi_).

Data derived from both clinical trials and real‐life practice suggest that most subjects with long‐lasting virological suppression maintain undetectable viraemia 48 to 96 weeks after switching to DRV_rtv_ monotherapy (mtDRV_rtv_). However, mtDRV_rtv_ is less effective than cART, as transient detectable viral loads (blips) are more frequent 5, 6, 7, 8. Since 2009, protease inhibitor (PI)‐based monotherapy is considered as a simplification option in both the Spanish and European guidelines for the use of antiretroviral agents in HIV‐1‐infected adults without history of failure on prior PI‐based therapy and who have had viral load <50 copies/mL for more than 6 months 9, 10. In the clinical practice, DRV_rtv_ is currently being replaced by DRV_cobi_, but there are no data about the effectiveness of mtDRV_cobi_ and whether a lower DRV C_trough_ could impacts on efficacy or an increase in the numbers of blips. The aim of this study was to evaluate the efficacy of mtDRV_cobi_ in the daily clinical practice, to analyse the relationship between pharmacokinetic parameters and virological failure, and to compare it with historical data on mtDRV_rtv_.

## 2 Materials and methods

This prospective observational study was carried out at the Virgen del Rocío University Hospitals in Spain. All subjects who maintained virological suppression ≥6 months and who switched to mtDRV_cobi_ once daily from January 2015 to January 2016 were included. Subjects with previous virological failure (VF) while on a PI‐containing regimen were included if the genotypic resistance tests showed no major (I47V, I50V, I54M/L, L76V and I84V) or ≤3 minor resistance mutations associated with reduced susceptibility to DRV according to the 2014 International AIDS Society criteria 11. The prescription of mtDRV_cobi_ was based on the criteria of the attending physicians as part of their daily clinical practice based on the encouraging results of several clinical trials 12, 13, 14, 15, 16, 17 and personal experience on boosted‐PI monotherapy 18, 19, 20, 21, 22, 23, 24, 25, 26, 27, 28, with the objective of avoiding toxicity associated with nucleoside analogues, increasing adherence, and to augment the cost‐effectiveness of therapy 29. Inclusion was not dependent on CD4^+^ T cell counts, hepatitis C virus (HCV) coinfection, laboratory parameters or the presence of viral blips during the previous 12 months.

In our hospital, mtDRV_cobi_ was not prescribed in case of pregnancy, hepatitis B coinfection or for concomitant use with drugs having potential adverse interactions with DRV_cobi_ pharmacokinetics 30. This study was conducted according to the principles of the Declaration of Helsinki and was approved by the Ethics Committee for Clinical Research of the Virgen del Rocío University Hospital. All subjects provided written informed consent to use their anonymized data and to perform plasma drug monitoring.

### 2.1 Endpoints, follow‐up and assessments

The primary clinical endpoint was treatment effectiveness, assessed as the percentage of subjects with virological suppression after 48 and 96 weeks according to intention‐to‐treat (ITT) analysis (non‐complete/missing = failure). Virological failure (VF) was defined as two consecutive confirmed plasma HIV‐RNA >200 copies/mL, or a single HIV‐RNA level >200 copies/mL if followed by a loss to follow‐up. A cut‐off level of 200 copies/mL was chosen as a more accurate measurement of VF since values <200 copies/mL suffer high variability and the risk of emerging resistance is believed to be relatively low 31, 32. An additional estimation of virological failure rates using 50 copies/mL as criteria for VF was made to compare with other studies. As a secondary outcome, virological efficacy was assessed using on‐treatment (OT) analysis, where subjects who discontinue therapy for any reason, as well as those who are lost to follow‐up, are not considered. In addition, a pharmacological sub‐study was performed in which the association between plasma levels and treatment outcome was analysed. As reference, efficacy data and pharmacological results were compared with those of a historical cohort of 150 subjects who started mtDRV_rtv_ at our centre 8.

Subject assessments were performed at baseline and every 3 months thereafter, including adherence (subject self‐report and pharmacy records), adverse events (AEs), biochemical and haematological profiles, flow cytometric counts of CD4^+^ T cells and plasma HIV‐RNA levels (COBAS AmpliPrep/COBAS TaqMan HIV‐1 test, version 2.0). AEs and abnormal laboratory findings were evaluated according to a standardized toxicity grade scale (AIDS Clinical Trials Group) 33. Genotypic resistance tests were performed on subjects with VF when viral load levels were sufficient. Subjects who missed two consecutive scheduled visits were considered lost to follow‐up.

### 2.2 Blood sampling and determination of DRV concentrations

Blood samples were drawn 24 h (±30 min) after the previous DRV_cobi_ dose taken after standard breakfast and processed within an hour after collection. Plasma was separated and stored at −80°C until assayed. Separation was performed on a Phenomenex Luna C18 (5 μm, 150 × 2.0 mm) analytical column. The mobile phase was composed of 2 mM ammonium acetate 0.1% formic acid and acetonitrile 0.1% formic acid. DRV was extracted from the plasma by protein precipitation, using acetonitrile containing a deuterated internal standard. Plasma DRV concentrations were determined using LC–MS/MS based on an adapted method 34 with standard curves that were highly linear over the range of 50 to 10,000 ng/mL and an intra‐ and inter‐assay precision and accuracy of <15%.

### 2.3 Statistical analysis

Categorical and quantitative variables were compared using the χ^2^ test, Student's t‐test or Mann‐Whitney nonparametric test, according to their distribution. Time‐to‐event analyses were performed by Kaplan‐Meier survival curves. Both the intra‐ and inter‐subject variability in drug concentrations was measured using the coefficients of variation (CV) of the available values from each subject. Pharmacokinetic data were compared with those of the historical DRV_rtv_ cohort 8, where 587 samples from 119 subjects were analysed. Statistical analyses were performed using the IBM software (SPSS v. 23.0, Chicago, USA), and *p*‐values <0.05 were considered significant.

## 3 Results and discussion

A total of 234 subjects were included in the study with a median follow‐up of 96 weeks (IQR, 58 to 96; range, 24 to 96). Baseline characteristics are described in Table 1. Before switching to mtDRV_cobi_, 175 (74.8%) subjects were on monotherapy as maintenance regimen (144 on DRV_rtv_, and 31 on LPV_rtv_), while 48 (20.5%) and 11 (4.7%) subjects were on triple and dual therapy, respectively.

**Table 1:** Baseline characteristics of the study population (n = 234)

Parameter	Value
Male, no. (%)	178 (76.1)
Age (years), M (IQR)	49.5 (43 to 54)
Weight (kg), M (IQR)	71.5 (62.5 to 83)
BMI kg/m^2^, M (IQR)	25 (22 to 27)
Nadir CD4^+^/μL, M (IQR)	150 (52 to 248)
CD4^+^ T cells/μL, M (IQR)	662 (512 to 837)
Zenith HIV‐RNA log_10_ copies/mL, M (IQR)	4.8 (4.1 to 5.3)
Previous CDC C stage, no (%)	66 (28.2)
Risk factor for HIV, no. (%)	
Previous intravenous drug use	96 (41)
Homosexual contact	62 (26.5)
Heterosexual contact	66 (28.2)
Other	10 (4.3)
Chronic hepatitis C, no. (%)	40 (17.1)
Cirrhosis no. (%)	8 (3.4)
Months on treatment, M (IQR)	141 (92 to 195)
Months with undetectable HIV‐RNA, M (IQR)	85 (50 to 119)
Presence of blips in the previous 12 months, no. (%)	22 (9.4)
Previous failure on protease inhibitors, n (%)	154 (65.8)
Previous ART regimens	
Monotherapy regimens	175 (74.8)
DRV_rtv_ monotherapy	144 (61.5)
LPV_rtv_ monotherapy	31 (13.2)
Dual therapy regimens	11 (4.7)
ATV + 3 TC	7 (2.9)
DRV_rtv_ + 3TC	1 (0.42)
Others	3 (1.28)
Triple therapy regimens	48 (20.5)
CKD‐EPI mL/min/1.73 m^2^, M (IQR)	99.1 (83.2 to 105.8)
CKD‐EPI < 60 mL/min/1.73 m^2^, no. (%)	3 (1.2)

M (IQR), Median (interquartile range), CDC, Centers of Disease Control; HCV, hepatitis C virus; ART, antiretroviral therapy; DRV_rtv_, ritonavir‐boosted darunavir; LPV_rtv_, ritonavir‐boosted lopinavir; ATV, atazanavir; 3TC, lamivudine; CKD‐EPI, Chronic Kidney Disease Epidemiology Collaboration.

One hundred and fifty‐four (65.8%) subjects had an earlier VF while receiving non‐boosted PI, including 30 (12.8%) subjects who had experienced a previous VF on a DRV_rtv_‐based regimen caused by treatment withdrawal. Genotypic resistance tests before switching to mtDRV_cobi_ was available for 127 subjects who had shown VF, including all 30 subjects who had failed to DRV_rtv_, showing no major resistance mutations to DRV in any case.

### 3.1 Efficacy and safety

**Figure 1:**
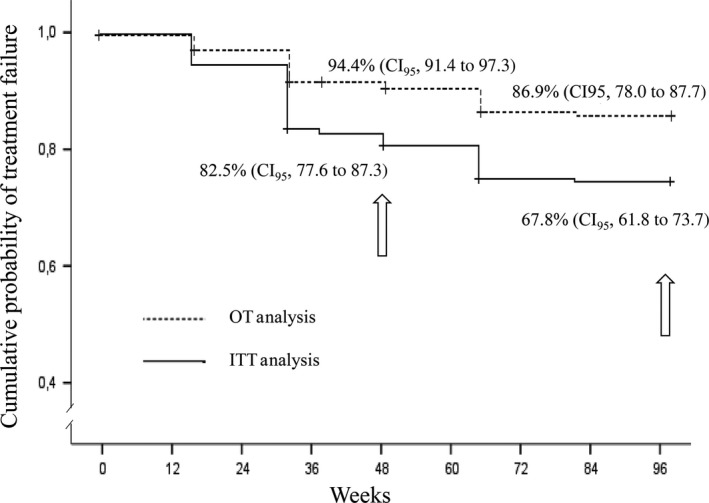
Kaplan‐Meier estimations of efficacy by on‐treatment (OT) and by intention‐to‐treat (ITT) at 48 and 96 weeks.

The Kaplan‐Meier estimations of treatment effectiveness by ITT analysis were 82.5% (CI_95_, 77.6 to 87.3) and 67.8% (CI_95_, 61.8 to 73.7) at week 48 and 96, respectively, while the historical control data with mtDRV_rtv_ were 82.7% (CI_95_, 76.7 to 88.7) and 67.6% (CI_95_, 60.0 to 75.2) respectively. In an OT analysis, the values in the present cohort were 94.4% (CI_95_, 91.4 to 97.3) and 86.9% (CI_95_, 78.0 to 87.7), respectively, and 94.4% (CI_95_, 91.4 to 97.3) and 83.6% (CI_95_: 77.2 to 90.0) in the historical cohort (Figure 1). The estimations of virological failure rates using 50 copies/mL as criteria for virological failure are displayed in Table 2.

**Table 2:** Kaplan‐Meier estimations of the efficacy of mtDRV_cobi_ using HIV‐RNA >50 copies/mL x2 or >50 x1 followed by loss to follow‐up as definition for virological failure

Virological failure definition	Virological efficacy	
	Week 48	Week 96
HIV‐RNA >200 copies/mL x2 or >200 x1 followed by loss to follow‐up	94.4% (CI_95_, 91.4 to 97.3)	86.2% (81.7 to 90.6)
HIV‐RNA >50 copies/mL x2 or >50 x1 followed by loss to follow‐up	88.9% (CI_95_: 84.8 to 92.9	81.4% (CI_95_: 76.4 to 86.3)

Twenty‐seven subjects (11.5%) had VF as defined by the protocol with a median plasma HIV‐RNA at failure of 1450 copies/mL (IQR, 244 to 5707). Eleven of these subjects were on cART before switching to monotherapy, 14 subjects were on monotherapy regimens and two on dual therapy.

There was no significant difference in the VF rates among those subjects with and without an earlier VF while on a PI‐based regimen (9.7% vs. 15.0% respectively; *p *=* *0.263). Furthermore, 60 (25.6%) subjects had a blip episode while on mtDRV_cobi_, whereas this figure was 27.6% in the historical cohort on mtDRV_rtv_ (*p *=* *0.713). After 6 months, 26 out of 27 subjects who experienced a VF regained virological control either by adding one (n* *=* *11) or two (n* *=* *8) NRTIs to mtDRV_cobi_ or switching to another triple therapy (n* *=* *8). Other treatment failures were due to AEs (n* *=* *7; grade 2 hypercholesterolaemia, 1; grades 1 and 3 hypertriglyceridaemia, 2; and grade 1 gastrointestinal disorder, 4), loss to follow‐up or treatment dropout (n* *=* *10), switching to another regimen by physician decision without VF criteria (n* *=* *6), death unrelated to treatment (n* *=* *1),and other reasons not related to the treatment, such as imprisonment, pregnancy, drug interactions or relocation (n* *=* *22). All these subjects had an undetectable viral load at the time of the last available HIV‐RNA assessment.

Regarding the CD4^+^ T cell counts, the mean increase from baseline to week 96 was 75 cells/mL (CI_95_, 25 to 125). Aminotransferase level elevations throughout the follow‐up occurred in 6/40 (15%) subjects with chronic hepatitis (grades 1, 5; grades 2, 1) and in 17/194 (8.8%) subjects without chronic hepatitis (grades 1, 12; grades 2, 2; grades 3, 1). All aminotransferase elevations were transient and improved without treatment modification. No significant changes were found in the lipid profiles. Overall, the mean changes in fasting total cholesterol and triglycerides in the subjects who completed 96 weeks were 5 mg/dl (CI_95_,−3 to 13) and −17 mg/dL (CI_95_, −42 to 7) (*p*‐values >0.1).

### 3.2 Pharmacokinetics of DRV

A total of 135 DRV determinations were performed in plasma samples derived from 83 subjects throughout the follow‐up period. The median of the plasma samples per subject was 2 (range, 1 to 2). Plasma DRV C_trough_ was lower for mtDRV_cobi_ (median, 1305 ng/mL; IQR, 652 to 2058; range, 150 to 5895) as compared to data of subjects on DRV_rtv_, (median, 1710 ng/mL; IQR, 1160 to 2210; range, 200 to 3838) (*p *=* *0.05). We did not observe relationships between plasma DRV concentrations and weight (*r* ^2^ = 0.106; *p *=* *0.340) or body mass index (*r* ^2^ = 0.127; *p *=* *0.282), nor differences in plasma DRV C_trough_ according to gender, the presence of chronic hepatitis, cirrhosis or renal dysfunction. The median intra‐subject variability was 27.4% (IQR, 18.5 to 54.0; range, 12.8 to 130.5) for subjects on mtDRV_cobi_, and 38.8% (IQR, 25.4 to 59.1; range, 3.7 to 213.1) for subjects on mtDRV_rtv_. Inter‐subject variability for subjects with more than one DRV C_trough_ determination (n = 57) was 92.1% for those on mtDRV_cobi_ and 43.6% for those on mtDRV_rtv_, respectively. We did not find any statistically significant difference between DRV plasma concentration and subjects with and without VF, median; 738 ng/mL (IQR; 467 to 2037) and 1378 ng/mL (IQR; 705 to 2185) (*p *=* *0.710) respectively.

Although somewhat less effective than triple therapy, more than 90% of virologically suppressed subjects switching to mtDRV_rtv_ maintained virological control in the clinical practice, even in subjects with previous VF‐ on PI‐based regimens when no major resistance mutations for DRV were present 7, 8. As it has been reported from various studies, monotherapy has a higher frequency of blips, although this has not been related to a higher frequency of VF. Moreover, in patients who failure on monotherapy with boosted PI, no resistance mutations have been found, and the introduction of analogues has been enough to control the infection again 35, 36, 37. These results, the benefits of regimens lacking the toxicity of nucleoside analogues, the low incidences and grades of the AEs, and to save up in antiretroviral drug costs 29 support the use of mtDRVrtv in clinical practice.

Recently, Ciaffi et al. reported a rate of virological failure as high as 21% at week 48 with DRV‐ or lopinavir‐based monotherapy as second‐line maintenance treatment for HIV‐1‐infected patients in sub‐Saharan Africa 38. Almost certainly these results were due to the fact that only 80% of the patients had a viral load <50 copies/mL at baseline.

Currently, DRV_rtv_ is being replaced by DRV_cobi_ in spite of an about 30% lower DRV C_trough_ as observed in healthy volunteers. To the best of our knowledge, this is the first study addressing the effectiveness of mtDRV_cobi_. Our results show that the DRV C_trough_ is about 22% lower for DRV_cobi_ than the concentrations observed from our historical data with mtDRV_rtv_. However, these concentrations remain above sixfold the protein binding‐adjusted EC_90_ for wild‐type HIV‐1 (200 ng/mL) and above threefold the protein binding‐adjusted EC_50_ for resistant HIV‐1 (550 ng/mL) 39 in most subjects. Two and 13 subjects had a C_trough_ below of 200 and 550 ng/mL, respectively, of whom only two subjects had VF, presenting a DRV C_trough_ of 463.82 and 353.83 ng/mL. While the intra‐subject variability in DRV C_trough_, was similar for both regimens, the inter‐subject variability appeared to be higher in the case of DRV_cobi_, although the number of subjects is insufficient to draw conclusions on this issue. Nonetheless, the effectiveness was similar to that observed previously with mtDRV_rtv_ 7, 8. The frequency of AEs in the present study was below 2%, which can in part be explained by the large proportion of subjects who were on a DRV_rtv_‐based regimen before switching to mtDRV_cobi_.

Our study has some limitations. First, blood samples for drug monitoring in the present study was not as frequent as in the earlier study, as in the last years the blood samples for the control of the HIV infection can be collected in primary health centres from where the samples are transferred in the same morning to our hospital. However, the analysis of pharmacokinetics was not the primary aim of the present work and still a considerably high proportion of patients representing the overall population could be analysed. Furthermore, although the number of subjects with VF are low, the data obtained from those subjects who were virologically suppressed show that the lower DRV concentrations observed when boosting with cobicistat appear not to impact on treatment outcome. Second, data from a previous cohort on mtDRV_rtv_ was used as reference, since the present study is a prospective observational study that does not include controls. Still, both data sets were obtained from one single cohort including subjects seen at one single centre and by the same physicians, following a unique protocol. Therefore, the data can be regarded as comparable.

## 4 Conclusion

According to the data obtained in our study, it seems that the change ritonavir to cobicistat does not affect the efficacy of monotherapy with DRV, regardless of the lower plasma C_trough_ achieved.

## Competing interests

L. F. Lopez‐Cortes, P. Viciana and N. Espinosa have received unrestricted research funding, consultancy fees, and lecture fees from and have served on the advisory boards of Abbott, Bristol‐Myers Squibb, Gilead Sciences, Janssen‐Cilag, Merck Sharp & Dohme, and ViiV Healthcare. The other authors have no conflicts of interest to disclose**.**

## Authors’ contributions

1. LFLC, AGV and MTR conceptualized and designed the study.
2. LFLC obtaining funding for the study.
3. LFLC, PV and NE contributed to provision of study materials or subjects.
4. AGV and TFM collected darunavir plasma levels.
5. AGV and MTR collected, assembled the data and managed the database.
6. AGV, MTR and LFLC analysed and interpreted the data.
7. AGV, MTR and LFLC drafted the article.
8. NE and PV critically reviewed the article for important intellectual content.
9. All authors approved the final version of the article:.
